# Association of Perineural and Lymphovascular Invasion with Clinical and Pathological T Staging in Head and Neck Squamous Cell Carcinoma

**DOI:** 10.3390/jcm15135317

**Published:** 2026-07-07

**Authors:** Aldona Chloupek, Paweł Grab, Dominika Zawadka-Modras, Dariusz Jurkiewicz

**Affiliations:** Department of Otolaryngology and Laryngological Oncology with Clinical Department of Cranio-Maxillofacial Surgery, Military Institute of Medicine—National Research Institute in Warsaw, 04-141 Warsaw, Poland; pgrab@wim.mil.pl (P.G.); dzawadka@wim.mil.pl (D.Z.-M.); djurkiewicz@wim.mil.pl (D.J.)

**Keywords:** head and neck squamous cell carcinoma, perineural invasion, lymphovascular invasion, pathological T stage, HNSCC

## Abstract

**Background/Objectives**: Perineural invasion (PNI) and lymphovascular invasion (LVI) are adverse histopathological features in head and neck squamous cell carcinoma (HNSCC). Their relationship with clinical and pathological T categories remains clinically relevant, particularly in surgically treated cohorts. This exploratory retrospective study evaluated the frequency of PNI and LVI according to clinical and pathological T stage in primary HNSCC staged according to the AJCC/UICC 8th edition. **Methods**: We analyzed 170 consecutive surgically treated patients with primary HNSCC. PNI and LVI were recorded from histopathology reports of resection specimens. **Results**: PNI status was available in 124 cases and was positive in 61 (49.2%); LVI status was available in 126 cases and was positive in 62 (49.2%). PNI frequency increased with advancing clinical T stage (12.5% in cT1, 54.3% in cT2, 70.0% in cT3, and 69.2% in cT4) and pathological T stage (14.3% in pT1, 51.5% in pT2, 71.9% in pT3, and 66.7% in pT4). LVI also increased with higher clinical T stage (28.1%, 53.2%, 57.1%, and 61.5%) and pathological T stage (25.7%, 50.0%, 54.6%, and 75.0%). Tongue tumors showed descriptively high crude proportions of PNI and LVI, but these subgroup findings were based on small numbers and were not adjusted for T stage, depth of invasion, or other confounders. **Conclusions**: In this single-center cohort, PNI and LVI were more frequent in higher T categories. These findings should be interpreted as descriptive and hypothesis-generating, because no recurrence, survival, or treatment-outcome analyses were available and the adjusted models were exploratory.

## 1. Introduction

Head and neck squamous cell carcinoma (HNSCC) is a clinically heterogeneous disease that includes tumors from distinct anatomical subsites with different etiologies, patterns of spread, and prognoses. It remains a major global health problem, accounting for roughly 4–5% of all malignancies and ranking among the ten most common cancers worldwide [1,2,3]. Despite advances in surgery, radiotherapy, and systemic therapy, 5-year overall survival for HNSCC has plateaued around 50%, largely because many patients present with locally advanced disease, and a significant proportion of early-stage tumors recur [4,5,6]. TNM stage remains central to treatment planning, but it does not fully capture pathological aggressiveness. Perineural invasion (PNI) and lymphovascular invasion (LVI) are adverse histopathological features associated with locoregional failure, distant metastasis, and reduced survival [7,8]. Their reporting is therefore important in postoperative risk assessment, especially when adjuvant treatment decisions are being considered.

Most data on PNI and LVI come from oral cavity squamous cell carcinoma (OCSCC), where depth of invasion (DOI), nodal metastasis, margin status, and extranodal extension are key determinants of outcome [8,9,10,11,12,13]. The AJCC/UICC 8th edition incorporates DOI into oral cavity T classification and extranodal extension into nodal staging, improving clinical relevance compared with earlier staging systems [14]. However, real-world surgical HNSCC series often include mixed subsites, and the extent to which crude PNI/LVI frequencies vary across T categories and locations is not always clearly reported. Because subsite, DOI, and stage are interrelated, location-specific findings must be interpreted cautiously unless adequately adjusted. A recent systematic review of HNSCC including more than 25,000 patients showed that PNI is present in approximately one third of cases and confers an approximately two-fold increased risk of death and recurrence compared with PNI-negative disease [7]. Lymphovascular invasion has emerged as a strong correlate of occult neck metastasis and the need for adjuvant therapy [9,10,11]. Emerging data indicate that the probability of detecting PNI-positive and LVI-positive disease is not uniform across the spectrum of head and neck tumors.

The aim of the present study was to describe, in an exploratory manner, the frequency of PNI and LVI across clinical and pathological T categories in a consecutive surgically treated HNSCC cohort, with additional descriptive reporting by primary tumor location. The study was not designed to establish independent prognostic value, to define treatment-changing thresholds, or to determine whether PNI/LVI remains independently associated with outcomes after adjustment for DOI, nodal status, HPV status, margins, extranodal extension, and adjuvant therapy.

## 2. Materials and Methods

### 2.1. Study Design and Patient Selection

This retrospective, single-center observational study was conducted at a tertiary referral institution (WIM-PIB). Consecutive patients surgically treated between 2022 and 2025 were included. Eligibility criteria were primary HNSCC, resection of the primary tumor, and histopathological confirmation of HNSCC. The cohort was not restricted to OCSCC because the aim was to describe a real-world surgical HNSCC population treated at the center; however, primary tumor site was recorded and all site-specific analyses were considered descriptive. Neck lymph node status was assessed and managed according to routine clinical practice, including clinical examination, imaging, and histopathological evaluation when neck specimens were available. Clinical and pathological data were obtained from electronic medical records and histopathology reports. Collected variables included primary tumor location, clinical and pathological T category, nodal status, DOI, PNI, and LVI. Clinical and pathological T and N categories were abstracted from contemporaneous-source clinical and pathology records generated during routine care in 2022–2025, when the AJCC/UICC 8th edition represented the current staging framework [14]. DOI was extracted where available, because it is a key component of modern oral cavity T classification. The study did not include independent central restaging of every case beyond the information contained in the original records; pathological staging was used for the primary analyses. Clinical and pathological T categories were analyzed separately because clinical T stage reflects preoperative assessment used for treatment planning, whereas pathological T stage incorporates postoperative microscopic information, including DOI in oral cavity tumors. PNI was considered present when tumor cells were identified within, around, or tracking along a nerve, whereas LVI was considered present when tumor cells were identified within endothelial-lined lymphatic or vascular spaces. Assessment was based on routine diagnostic histopathology reports of resection specimens. Regional lymph node metastasis was determined based on standard clinical assessments, including histopathology when nodal specimens were available, computed tomography (CT), and clinical examination. Depth of invasion was assessed on the resection specimen during histopathological examination. Ethics approval was not required because, under Polish law, retrospective research based on the analysis of medical records is not considered a medical experiment.

### 2.2. Statistical Analysis

Categorical variables were summarized as *n*/*N* (%), and continuous variables as mean and standard deviation (SD) and/or median and interquartile range (IQR) or range. Analyses used available-case data for each comparison. No imputation was performed. Associations between PNI or LVI and clinical/pathological T stage (T1–T4) were assessed with Pearson’s chi-square (χ^2^) test, with effect size reported as Cramér’s V. Logistic regression with T stage entered as an ordinal predictor provided odds ratios (OR) per one-stage increase (95% CI). Trends across ordered T categories were tested using the Cochran–Armitage test. Clinical and pathological T stages were analyzed separately to distinguish preoperative clinical assessment from definitive postoperative staging. Comparisons by tumor location were descriptive only due to sparse cell counts and lack of adjustment for T stage or DOI. Fully adjusted prognostic analyses incorporating HPV status, margin status, extranodal extension, adjuvant therapy, recurrence, and survival outcomes were not performed because these variables were not uniformly available. The multivariable models were therefore limited to variables available in the database and were considered exploratory. All tests were two-sided, with α = 0.05. Calculations were performed using STATISTICA 13.3. Additional sensitivity analyses were performed using the source database. Baseline characteristics were compared between patients with and without evaluable PNI/LVI status to assess potential selection bias. Sensitivity analyses were repeated after restricting the cohort to OCSCC. Exploratory multivariable logistic regression models were also fitted using complete-case data, with pathological T category entered as an ordinal predictor and adjustment for DOI, pathological nodal positivity, and tongue versus non-tongue site. These adjusted analyses were considered hypothesis-generating because of the limited number of complete cases and events per variable.

## 3. Results

### 3.1. Study Population and Tumor Characteristics

A total of 170 patients with HNSCC were included in the analysis. There were 101 men (59.4%) and 69 women (40.6%). The median age at surgery was 62 years (IQR: 54–70; range 36–96 years; mean 61.9 (12.3) years). Tumors most frequently originated in the tongue, which accounted for more than half of all cases (54.7%), while the floor of the mouth represented approximately one-fifth (22.4%). Less common primary sites included the maxilla (8.8%), skin (7.6%), lip (4.7%), and lateral pharyngeal wall (1.8%). At presentation, most patients had intermediate clinical tumor stages, with cT2 and cT1 lesions being most frequent (43.5% and 27.1%, respectively), whereas advanced clinical T3 and T4 tumors were less common. A similar distribution was observed for pathological T stage; T1 and T2 comprised roughly two-thirds of the cohort (32.9% and 30.6%, respectively), with pathological T3 and T4 accounting for 22.4% and 14.1% of patients, respectively. With respect to nodal status, approximately two-thirds of patients were clinically node-negative (cN0), while the remaining third presented with varying degrees of nodal involvement (≥cN1). A broadly similar pattern was seen on histopathology: around one-third had neck metastases confirmed in histopathology examination, predominantly in lower-burden categories (pN1–pN2b). Data on the DOI were available in 131 of 170 patients (77.1%). The median DOI was 6.0 mm (IQR: 3.0–10.5 mm; range 1–50 mm, mean [SD]: 8.3 [7.9] mm). Detailed distributions of clinical and pathological L and N staging are provided in Table 1.

PNI status was available for 124 of 170 patients (72.9%). Sixty-one patients (49.2%) were PNI-positive. LVI was assessable in 126 patients (74.1%), and 62 patients were LVI-positive (49.2%).

### 3.2. Perineural Invasion and T Stage

Perineural invasion was strongly associated with both clinical and pathological T stage. With increasing clinical T category, the proportion of PNI-positive tumors rose from a low level in early-stage disease to approximately two-thirds in advanced stages (Table 2). A significant association between PNI and clinical T stage was observed (χ^2^(3) = 25.37, *p* < 0.0001, Cramér’s V = 0.45; χ^2^ test of independence). In univariable binary logistic regression with T category modeled as an ordinal predictor, each one-stage increase in clinical T was associated with more than a twofold increase in the odds of PNI (OR per stage = 2.27, 95% CI 1.54–3.35, *p* < 0.0001).

A similar pattern was observed for pathological T stage (Table 2). Across pathological T categories, the proportion of PNI-positive tumors increased from 14.3% in pathological T1 to 71.9% in pathological T3 and remained high in pathological T4 (66.7%). We also measured the association between pathological T stage and PNI (χ^2^(3) = 26.65, *p* < 0.0001, Cramér’s V = 0.46; χ^2^ test of independence). In univariable binary logistic regression with T category modeled as an ordinal predictor, each one-stage increase in pathological T stage was associated with more than a twofold increase in the odds of PNI (OR per stage = 2.34, 95% CI 1.59–3.43, *p* < 0.0001).

### 3.3. Lymphovascular Invasion and T Stage

The proportion of LVI-positive tumors increased with higher clinical T stages, from 28.1% in cT1 to 61.5% in cT4 (Table 3). A small-to-moderate association between LVI status and clinical T stage was observed (χ^2^(3) = 8.10, *p* = 0.0440, Cramér’s V = 0.25; χ^2^ test of independence). In univariable binary logistic regression with T category modeled as an ordinal predictor, each one-stage increase in clinical T was associated with a 53% increase in the odds of LVI (OR per stage = 1.53, 95% CI 1.09–2.16, *p* = 0.0149).

The proportion of LVI-positive tumors also increased with higher pathological T stage from 25.7% in pT1 to 75.0% in pT4 (Table 3). A moderate association between LVI status and pathological T stage was observed (χ^2^(3) = 14.50, *p* = 0.0023, Cramér’s V = 0.34; χ^2^ test of independence). In univariable binary logistic regression with T category modeled as an ordinal predictor, each one-stage increase in pathological T was associated with a 92% increase in odds of LVI (OR per stage = 1.92, 95% CI 1.34–2.74, *p* = 0.0004).

### 3.4. Perineural Invasion and Tumor Location

PNI varied descriptively by tumor location (Table 4). PNI was most frequently observed in tongue tumors, whereas lower proportions were seen in lip and maxillary tumors. No PNI cases were recorded in the lateral pharyngeal wall subgroup. Because several location categories contained very small numbers, resulting in sparse data and low expected cell counts, no inferential comparison of PNI across tumor locations was performed. Therefore, the apparently higher crude PNI proportion in tongue cancer should not be interpreted as evidence of a site-specific effect independent of T category, DOI, or other adverse pathological variables.

### 3.5. Lymphovascular Invasion and Tumor Location

A similar descriptive pattern was observed for LVI (Table 5). LVI appeared most frequently in tongue and maxillary tumors, at an intermediate rate in floor-of-mouth tumors, and relatively uncommonly in lip tumors. No LVI cases were observed in the small lateral pharyngeal wall subgroup. As with PNI, sparse counts across several tumor location categories limited the validity of asymptotic testing. Therefore, no reliable global comparison of LVI across locations was performed, and the results are reported descriptively rather than as site-adjusted or stage-adjusted estimates.

### 3.6. Trends in the Proportion of PNI- and LVI-Positive Tumors with Advancing T Stage

The Cochran–Armitage test confirmed a significant increasing trend in the proportion of PNI- and LVI-positive tumors with advancing clinical T and pathological T stage. For PNI, a significant increasing trend across T categories was observed for both clinical T stage (χ^2^_trend(1) = 19.37, *p* = 1.1 × 10^−5^) and pathological T stage (χ^2^_trend(1) = 21.18, *p* = 4.2 × 10^−6^). A similar significant increasing trend was observed for LVI across both clinical T stage (χ^2^_trend(1) = 6.12, *p* = 0.013) and pathological T stage (χ^2^_trend(1) = 13.60, *p* = 2.3 × 10^−4^).

### 3.7. Sensitivity and Adjusted Analyses

PNI status was unavailable in 46/170 patients (27.1%) and LVI status was unavailable in 44/170 patients (25.9%). Missingness was not completely random: patients without evaluable PNI/LVI data had smaller tumors and lower T categories. For PNI, median tumor size was 34.5 mm in evaluable cases versus 22.5 mm in non-evaluable cases (*p* = 0.002), and advanced pathological T3-T4 disease was present in 56/124 evaluable cases (45.2%) versus 6/46 non-evaluable cases (13.0%; *p* < 0.001). Similar differences were observed for LVI: median tumor size was 34.5 mm versus 21.5 mm (*p* = 0.001), and pathological T3-T4 disease was present in 57/126 evaluable cases (45.2%) versus 5/44 non-evaluable cases (11.4%; *p* < 0.001). These findings support the need to interpret available-case analyses cautiously.

When analyses were restricted to OCSCC (*n* = 154; 108 evaluable for PNI and 110 evaluable for LVI), the ordered increase in PNI and LVI across T categories remained evident. For pathological T stage, PNI increased from 14.7% in pT1 to 66.7% in pT4 (OR per one-stage increase = 2.34, 95% CI 1.56–3.52, *p* < 0.001), while LVI increased from 26.5% in pT1 to 76.2% in pT4 (OR per one-stage increase = 2.07, 95% CI 1.40–3.05, *p* < 0.001).

Exploratory complete-case multivariable models were then fitted with pathological T stage, DOI, pathological nodal positivity, and tongue site (Table 6). For PNI (*n* = 90; 46 events), the association with pathological T stage was attenuated after adjustment (OR = 1.74 per stage, 95% CI 0.98–3.06, *p* = 0.057), whereas pathological nodal positivity remained strongly associated with PNI (OR = 12.42, 95% CI 3.67–42.08, *p* < 0.001). For LVI (*n* = 91; 43 events), pathological T stage remained associated with LVI after adjustment (OR = 2.08 per stage, 95% CI 1.21–3.59, *p* = 0.008), as did pathological nodal positivity (OR = 2.91, 95% CI 1.02–8.25, *p* = 0.045). In both models, DOI and tongue site did not reach statistical significance after adjustment. These analyses are exploratory and should not be interpreted as definitive prognostic models.

## 4. Discussion

Our study aimed to assess how PNI and LVI, two adverse histopathological features in HNSCC, are distributed across clinical and pathological T categories and across primary tumor sites in a real-world surgical cohort. The principal observation was that both features became more frequent with increasing local tumor extent. This finding is biologically plausible and consistent with existing literature, but it should be interpreted as a descriptive clinicopathological association rather than evidence that T category independently determines PNI or LVI. The value of the present analysis is therefore not to redefine the prognostic role of PNI or LVI, but to provide a transparent stage-stratified, site-aware, available-case description of their distribution in routine surgical practice, complemented by an OCSCC-only sensitivity analysis, missing-data assessment, and limited adjusted models incorporating DOI, nodal status, and tumor site. The strong association between PNI and T stage is consistent with the contemporary evidence base in OCSCC and HNSCC. A large systematic review and meta-analysis that focused on OCSCC (101 studies; >26,000 patients) demonstrated that PNI was associated with inferior survival outcomes and higher recurrence risk [15]. More recently, Chiari et al. reported poor survival and oncologic outcomes in oral cavity adenosquamous carcinoma, with recurrence and prognosis influenced by adverse pathological characteristics including PNI, LVI, and margin status [16]. Similarly, a 2024 meta-analysis across HNSCC cases (74 studies; 27,559 patients) reported that PNI was associated with significantly worse survival and increased risks of local, locoregional, and distant failure, emphasizing that PNI-positive disease tends to cluster with more advanced and biologically aggressive presentations [7]. As tumors progress from early to advanced local categories, the probability of detecting PNI rises substantially, which is biologically plausible because deeper and more infiltrative tumors have a greater possibility of perineural spread and extension along neural sheaths. Cohort studies also support this coupling between PNI and more advanced clinicopathologic profiles. In a recent retrospective OCSCC study examining PNI as an independent prognostic factor for local recurrence, Saipooja et al. [17] found that PNI correlated with nodal metastasis, DOI, and stage, even when its independent relationship with local recurrence was less consistent after multivariable adjustment. This aligns with our findings: the strong association between PNI and T category probably reflects both increasing biological aggressiveness with stage and shared variance with other invasive-front features, such as DOI and nodal disease. Thus, PNI should not be viewed merely as an incidental microscopic finding. It likely reflects a biologically aggressive invasive pattern that often coexists with deeper local extension, nodal involvement, and other adverse pathological features. Our data add a focused available-case description of how frequently PNI is reported across clinical and pathological T categories in a real-world surgical cohort, but they do not establish its independent prognostic value within the same T category.

For LVI, we observed a statistically significant but more modest increase with advancing T category. This pattern is also consistent with current evidence indicating that LVI is closely linked to lymphatic dissemination, occult or overt nodal disease, locoregional recurrence, and inferior outcomes in oral cancer and HNSCC [10,11,18]. The recent systematic review by Chiari et al. [16] further supports this concept, showing that LVI, together with PNI and other adverse pathological factors, may contribute to unfavorable oncologic outcomes in oral cavity malignancy. In contrast to PNI, which is anatomically related to neural tracking and local infiltrative growth, LVI may more directly reflect access to lymphatic or vascular channels and therefore regional or systemic dissemination potential. This distinction may explain why the stage gradient for LVI was less pronounced than for PNI while still remaining clinically meaningful. Because recurrence and survival outcomes were not available, our study cannot test these prognostic associations directly; however, our findings are compatible with the view that LVI forms part of a wider adverse pathological profile. The stronger associations observed for pathological rather than clinical T stage should be interpreted cautiously. This difference is expected, because pathological staging incorporates postoperative microscopic information that is unavailable or only approximated before surgery. Recent studies have documented clinically meaningful discrepancies between clinical and pathological staging and have highlighted the management implications of upstaging after histopathological examination [19,20]. Multiple contemporary publications emphasize the central role of DOI in modern oral cavity staging and prognosis, showing that DOI can alter T staging and is associated with adverse pathological features, including PNI [21,22]. Moreover, a prospective study comparing radiologic, macroscopic, and microscopic DOI reported overall strong correlations but systematic overestimation in smaller lesions and noted that higher-risk pathological features such as PNI and LVI track with greater DOI and improved agreement between measurement modalities, reinforcing that invasive biology and deeper extension are intertwined and most accurately captured after resection [23]. These data provide a coherent explanation for why pathological T-based associations in our cohort were at least as strong as, and in some analyses stronger than, clinical T-based associations: pathological T more closely reflects the anatomic and microscopic substrate in which PNI and LVI are identified.

Location-stratified analyses by tumor subsite were limited to descriptive interpretation because missing data and sparse subgroup counts precluded robust inferential comparisons for both PNI and LVI. Any apparent location-specific differences should be interpreted with caution. However, the descriptive tendency toward higher frequencies of PNI and LVI in tongue tumors is compatible with other studies showing that these features are prognostically relevant in tongue squamous cell carcinoma and often coexist with advanced clinical stage, DOI, and nodal involvement. For example, in tongue squamous cell carcinoma, both immunohistochemistry-based and conventional pathology studies have identified PNI and LVI as adverse factors associated with poorer survival, with some evidence that combined positivity identifies a particularly high-risk subgroup [24,25]. Although our dataset does not allow definitive subsite comparisons or adjustment for all relevant covariates within each location, the directional agreement with these cohorts supports further investigation in larger, adequately powered datasets.

To address cohort heterogeneity, we performed an additional OCSCC-only sensitivity analysis. This analysis was important because the full cohort included biologically distinct HNSCC locations, including oral cavity and non-oral sites. The OCSCC-only analysis showed a similar direction of association between T category and PNI/LVI, which supports the internal consistency of the principal observation. Nevertheless, the mixed-cohort design remains a limitation. Tumors of the oral cavity, lip, skin, maxilla, and pharyngeal wall differ in etiology, local anatomy, staging considerations, and treatment pathways. Therefore, the overall HNSCC analysis should be viewed as a broad real-world description, whereas OCSCC-specific and subsite-specific conclusions require larger dedicated cohorts.

Taken together, the observed stage gradients are coherent with contemporary models of oral cavity tumor invasion. As tumors expand and penetrate deeper compartments, opportunities for both neural tracking and lymphovascular permeation increase, which provides a straightforward anatomic basis for rising frequency of PNI and LVI with increasing T category. Beyond anatomy, modern cancer neuroscience frameworks propose that tumor–nerve interactions can form an active microenvironmental niche that promotes invasion and treatment resistance, rather than PNI representing only passive spread along pre-existing structures [26]. This concept strengthens the biological rationale for why PNI prevalence rises with advancing local extent and why PNI is repeatedly associated with recurrence and survival across meta-analyses and cohorts [7,15]. Our findings position PNI and LVI as stage-associated markers of invasive tumor behavior rather than as isolated histological observations. The present study contributes a transparent stage-stratified and site-aware description from routine surgical practice, supported by sensitivity analyses restricted to OCSCC, assessment of missingness, and limited adjusted models incorporating DOI, nodal status, and tumor site. At the same time, the data do not establish independent prognostic value or treatment-modifying utility. The results should therefore be interpreted as a basis for hypothesis generation and for more standardized reporting, rather than as evidence sufficient to change adjuvant-treatment decisions.

The sensitivity and adjusted analyses clarified several methodological issues. PNI status was available in 124 patients and LVI status in 126 patients, leaving approximately one quarter of the cohort without evaluable documentation for these variables. The comparison of cases with and without available PNI/LVI information indicated that missingness was associated with smaller tumor size and lower T category. This suggests possible selection bias, because more advanced tumors may have more extensive sampling or more detailed reporting of adverse pathological features. The exploratory multivariable models also showed that pathological T category, DOI, nodal status, and tumor site are interrelated. After adjustment, the association between pathological T category and PNI was attenuated, whereas the association with LVI was more persistent. These models should not be overinterpreted because of the limited sample size and number of complete cases, but they appropriately temper the conclusion that PNI and LVI provide independent information in this dataset.

Clinically, PNI and LVI are important because they are frequently considered during postoperative risk assessment and adjuvant-treatment discussions. This clinical relevance is supported by recent multicenter evidence from Fermi et al. [27], who showed that adverse pathological characteristics, including PNI, extranodal extension, advanced nodal disease, and adjuvant treatment variables, were associated with survival outcomes in head and neck cutaneous squamous cell carcinoma with nodal metastases. The present study was not designed to determine whether PNI should independently prompt adjuvant radiotherapy in otherwise comparable patients, whether PNI or LVI retains independent prognostic value specifically in T1/T2 disease, or whether a combined model incorporating DOI, extranodal extension, margin status, PNI, and LVI improves risk stratification. Those questions require survival endpoints, standardized pathological assessment, and adequately powered multivariable models. Our findings instead support careful and consistent reporting of PNI and LVI and reinforce the need to interpret these features alongside T category, DOI, nodal status, margin status, HPV status where applicable, and postoperative treatment variables.

Several limitations should be considered. First, this was a retrospective single-center study with 170 patients, and PNI/LVI status was not evaluable in all cases. Second, the cohort combined several HNSCC subsites, which limits biological homogeneity and reduces the strength of subsite-specific conclusions. Third, HPV status, margin status, extranodal extension details, adjuvant therapy, recurrence, and survival outcomes were not uniformly available. Fourth, PNI and LVI were abstracted from routine diagnostic histopathology reports, and the database did not contain standardized case-level information on the use of ancillary stains. Fifth, the multivariable models were exploratory and complete-case only; therefore, they should be viewed as sensitivity analyses rather than definitive evidence of independence. Despite these limitations, the present analyses provide a transparent and clinically cautious description of how PNI and LVI are distributed across T categories in surgically treated HNSCC, with additional support from an OCSCC-only sensitivity analysis.

## 5. Conclusions

In this retrospective single-center cohort of surgically treated patients with HNSCC, both PNI and LVI were more frequently observed in higher clinical and pathological T categories. The primary association was descriptive and univariable, and stronger pathological-stage associations were expected because pathological staging captures postoperative microscopic information more completely than clinical staging. After adjustment in exploratory models, pathological T stage remained associated with LVI but was attenuated for PNI, underscoring that the main findings should be interpreted as descriptive and hypothesis-generating rather than as proof of independent prognostic value.

Tongue tumors showed descriptively high crude proportions of PNI and LVI, but these findings were based on small subgroup numbers and were not adjusted for T category, DOI, or other confounders. Therefore, they should not be interpreted as evidence of a site-specific effect. Overall, our findings support careful reporting of PNI and LVI as adverse pathological features associated with increasing tumor extent, while acknowledging that independent prognostic or treatment-modifying value was not demonstrated in this dataset.

Future studies should validate these observations in larger multicenter cohorts, preferably with OCSCC-specific and subsite-specific analyses, standardized histopathological definitions, complete HPV, DOI, margin, nodal, extranodal-extension, and adjuvant-treatment data, and survival or recurrence outcomes. Such studies are needed to determine whether PNI/LVI adds independent prognostic value in T1/T2 disease and whether models combining DOI, ENE, PNI, and LVI improve risk stratification.

## Figures and Tables

**Table 1:** Baseline clinical and pathological characteristics of the study cohort (*N* = 170).

Characteristic	Value
Sex, *n* (%):	
Male	101 (59.4)
Female	69 (40.6)
Age at surgery [years]:	
Mean (SD)	61.9 (12.3)
Median (IQR)	62 (54–70)
Primary tumor site, *n* (%):	
Tongue	93 (54.7)
Floor of mouth	38 (22.4)
Maxilla	15 (8.8)
Skin	13 (7.6)
Lip	8 (4.7)
Lateral pharyngeal wall	3 (1.8)
Perineural invasion	61 (49.2) ^a^
Lymphovascular invasion	62 (49.2) ^b^
Clinical T stage, *n* (%):	
T1	46 (27.1)
T2	74 (43.5)
T3	24 (14.1)
T4	26 (15.3)
Histopathological T stage, *n* (%):	
T1	56 (32.9)
T2	52 (30.6)
T3	38 (22.4)
T4	24 (14.1)
Clinical N stage, *n* (%):	
N0	115 (67.6)
N1	25 (14.7)
N2	3 (1.8)
N2a	2 (1.2)
N2b	15 (8.8)
N2c	8 (4.7)
N3	2 (1.2)
Histopathological N stage, *n* (%):	
N0	95 (56.5)
N1	22 (12.9)
N2a	2 (1.2)
N2b	15 (8.8)
N2c	6 (3.5)
N3	1 (0.6)
N3b	13 (7.6)
N3c	2 (1.2)
Nx	13 (7.6)
Depth of invasion [mm]:	
Mean	8.3 ± 7.9
Median (IQR)	6.0 (3.0–10.8)
Available	131 (77.1%)

Abbreviations: IQR, interquartile range; *N*, number of evaluable patients for the variable; *n*, number of patients in the category; SD, standard deviation. ^a^ Perineural invasion status was available in 124 cases. ^b^ LVI status was available in 126 cases.

**Table 2:** Perineural invasion by clinical and pathological T stage.

**Clinical T Stage (*N* = 124)**	**PNI Status Available (*n*)**	**PNI-Positive (*n*)**	**PNI-Positive (%)**
T1	32	4	12.5
T2	46	25	54.3
T3	20	14	70.0
T4	26	18	69.2
**Pathological T Stage (*N* = 124)**	**PNI Status Available (*n*)**	**PNI-Positive (*n*)**	**PNI-Positive (%)**
T1	35	5	14.3
T2	33	17	51.5
T3	32	23	71.9
T4	24	16	66.7

Abbreviations: *N*, number of evaluable patients for the variable; *n*, number of patients in given category.

**Table 3:** Lymphovascular invasion by clinical and pathological T stage.

**Clinical T Stage (*N* = 126)**	**LVI Status Available (*n*)**	**LVI-Positive (*n*)**	**LVI-Positive (%)**
T1	32	9	28.1
T2	47	25	53.2
T3	21	12	57.1
T4	26	16	61.5
**Pathological T Stage (*N* = 126)**	**LVI Status Available (*n*)**	**LVI-Positive (*n*)**	**LVI-Positive (%)**
T1	35	9	25.7
T2	34	17	50.0
T3	33	18	54.6
T4	24	18	75.0

Abbreviations: LVI, lymphovascular invasion; *N*, number of evaluable patients for the variable; *n*, number of patients in given category.

**Table 4:** Perineural invasion by tumor location (*N* = 124).

Tumor Location	PNI Status Available (*n*)	PNI-Positive (*n*)	PNI-Positive (%)
Lateral pharyngeal wall	3	0	0
Floor of mouth	38	17	44.7
Tongue	50	30	60.0
Skin	13	6	46.2
Maxilla	12	5	41.7
Lip	8	3	37.5

Abbreviations: *N*, number of evaluable patients for the variable; *n*, number of patients in given category; PNI, perineural invasion.

**Table 5:** Lymphovascular invasion by tumor location (*N* = 126).

Tumor Location	LVI Status Available (*n*)	LVI-Positive (*n*)	LVI-Positive (%)
Lateral pharyngeal wall	3	0	0.0
Floor of mouth	38	17	44.7
Tongue	51	33	64.7
Skin	13	4	30.8
Maxilla	13	7	53.8
Lip	8	1	12.5

Abbreviations: LVI, lymphovascular invasion; *N*, number of evaluable patients for the variable; *n*, number of patients in given category.

**Table 6:** Exploratory multivariable logistic regression models for PNI and LVI.

Outcome/Model	Complete Cases	Predictor	Adjusted OR	95% CI	*p* Value
PNI	*n* = 90; 46 events	Pathological T stage (per category)	1.74	0.98–3.06	0.057
PNI	*n* = 90; 46 events	Pathological nodal positivity	12.42	3.67–42.08	<0.001
PNI	*n* = 90; 46 events	DOI (per mm)	1.04	0.97–1.12	0.276
PNI	*n* = 90; 46 events	Tongue site	3.14	0.99–9.99	0.052
LVI	*n* = 91; 43 events	Pathological T stage (per category)	2.08	1.21–3.59	0.008
LVI	*n* = 91; 43 events	Pathological nodal positivity	2.91	1.02–8.25	0.045
LVI	*n* = 91; 43 events	DOI (per mm)	1.06	0.99–1.14	0.093
LVI	*n* = 91; 43 events	Tongue site	2.22	0.78–6.31	0.135

Abbreviations: CI, confidence interval; DOI, depth of invasion; LVI, lymphovascular invasion; OR, odds ratio; PNI, perineural invasion. Models used complete-case data and are exploratory.

## Data Availability

Data are available upon request from the corresponding author.

## Abbreviations

The following abbreviations are used in this manuscript:

AJCC	American Joint Committee on Cancer
CT	computed tomography
DOI	depth of invasion
HNSCC	head and neck squamous cell carcinoma
IQR	interquartile range
LVI	lymphovascular invasion
NVI	neurovascular invasion
OCSCC	oral cavity squamous cell carcinoma
OR	odds ratio
PNI	perineural invasion
SD	standard deviation
